# A Plea for a Paradigm Shift from X-Ray to Ultrasound in Adults: An Update for Emergency Physicians, General Practitioners, Orthopedists and Sports Medicine Physicians

**DOI:** 10.3390/diagnostics15141827

**Published:** 2025-07-21

**Authors:** Joseph Osterwalder, Beatrice Hoffmann, Mike Blaivas, Rudolf Horn, Eric Matchiner, Christoph F. Dietrich

**Affiliations:** 1 Executive Board, Polipraxis Group, 9000 St. Gallen, Switzerland; jo.osterwalder@outlook.com; 2 Department of Emergency Medicine, Beth Israel Deaconess Medical Center, Harvard Medical School, Boston, MA 02215, USA; bhoffma2@bidmc.harvard.edu; 3 Department of Medicine, School of Medicine, University of South Carolina, Columbia, SC 29605, USA; mike@blaivas.org; 4 Center da sandà Val Müstair, 7536 Sta. Maria, Switzerland; rudolf@horn-ch.org; 5 Bürgerspital Solothurn, 4500 Solothurn, Switzerland; eric.matschiner@posteo.de; 6 University Hospital Frankfurt, Johann-Wolfgang-Goethe University Frankfurt, Theodor-Stern-Kai 7, D-60596 Frankfurt am Main, Germany

**Keywords:** ultrasound imaging, bone fractures, diagnostic accuracy, guideline

## Abstract

This update is aimed at various specialists who deal with fractures, such as emergency physicians, general practitioners, orthopedists, and sports medicine physicians. The Global Burden of Disease 2019 Fracture Collaborators estimated the worldwide incidence to be at 178 million, i.e., 2.2 fractures per 1000 people per year. Traditionally, X-rays are the first choice for suspected fractures. However, many fractures can also be detected or excluded with ultrasound. This option is especially attractive when available at the “point of care,”, i.e., at the patient’s bedside in the ambulatory or emergency setting. Point-of-care ultrasound provides clinicians with a simple, cost-effective imaging tool without radiation and complex infrastructure. The evidence suggests that ultrasound has high diagnostic sensitivity and can reliably rule out many fractures with a high degree of certainty. When applied correctly, it could potentially save millions of radiographs and, in some cases, even compete with the accuracy of X-rays and CT scans. These findings suggest a potential paradigm shift. This update discusses the advantages of ultrasound, its examination technique, sonoanatomy of fractures, and relevant indication groups, including its application for analgesia through nerve, fascia, and fascial plane blocks. Ultrasound’s diagnostic value supports its integration into routine fracture assessment, particularly in emergency and ambulatory care settings

## 1. Introduction

In routine medical practice, conventional X-ray is the standard first-line imaging modality for fracture diagnosis. However, point-of-care ultrasound (PoCUS) is increasingly being recognized as a valuable tool for assessing fractures in emergency or ambulatory care settings. Clinicians from various specialties, such as family medicine, internal medicine, emergency medicine, hospitalists, orthopedists, podiatrists, and sports medicine practitioners, use ultrasound to diagnose fractures. Studies have demonstrated that ultrasound offers high diagnostic accuracy for certain fractures in many anatomical regions and, in some cases, rival X-rays and occasionally even CT scans, as it may even exceed their accuracy [1]. While the technique itself is simple, achieving optimal results requires expertise from the examiner. Currently, there is a lack of formal training curricula for bone fracture management, presenting a significant opportunity to expand clinicians’ capabilities. For physicians managing fractures, musculoskeletal (MSK) ultrasound provides substantial advantages, particularly for evaluating subtle fractures, as well as for assessing soft tissue injuries such as hematomas, joint effusions, and ligament and tendon injuries. These structures should always be considered in the comprehensive PoCUS evaluation if a fracture is suspected.

There are many reasons to choose ultrasound over conventional radiography. Unlike X-ray, which is limited by the summation effect of overlapping structures, ultrasound provides real-time visualization of boney surfaces and surrounding soft tissues in multiple planes. Moreover, ultrasound does not require a fixed or standardized position of the body part being examined. This feature allows clinicians to conduct the examination with the patient in the most pain-free position by moving the probe around the injured limb or body part.

MSK ultrasound offers a safe, rapid, and effective initial evaluation and, for some conditions, can serve patient comfort with minimal radiation exposure and can be considerably less costly [1]. However, as wave frequencies used in diagnostic ultrasound do not penetrate bone, the technique is limited to visualizing the cortical surface and does not allow for an overview of the anatomical condition of fractures. Further imaging may be necessary to assess intraosseous pathology and for a precise assessment of fractures.

Based on the current literature, numerous but often small studies have supported the clinical value of fracture ultrasound [1,2]. Data shows that it is safe and easy to perform, offers important advantages over X-rays, and, when properly used, saves time and reduces the need for additional CT and MRI studies. Despite these benefits, fracture and associated MSK ultrasound remains underutilized.

This article reviews and summarizes the clinical context, indications, and benefits of fracture ultrasound and provides recommendations for its rational application. Our goal is to inform a broader audience of physicians on how fracture ultrasound can be applied effectively in daily practice and in many instances replace X-ray.

For this purpose, we have divided the article into nine sub-topics:

1. Objectives;
2. Indications;
3. Sonographic signs of normal anatomy and fractures;
4. Examination technique;
5. Systematic workflow of ultrasound application in fracture diagnostics and regional pain therapy;
6. Limitations and future directions;
7. Narrative and visual reviews;
8. Clinical case examples—advantages and limitations of ultrasound compared to X-Ray and CT;
9. Conclusion.

This update does not focus on anatomical regions but focuses instead on indications for which there is strong evidence and potential practical use for fracture ultrasound.

## 2. Objectives

The use of fracture ultrasound in emergency situations aims to:

- Reduce reliance on X-rays;
- Promote a more rational use of X-rays, CTs and MRIs;
- Reduce ionizing radiation exposure;
- Reduce costs;
- Optimize personnel and infrastructural resources;
- Shorten the length of stay in the emergency department and other waiting areas;
- Improve diagnostic accuracy;
- Increase patient comfort;
- Use in algorithms as, for example, the Ottawa Rule.

## 3. Indications

Indications for fracture ultrasound in hospital-based emergency departments and outpatient settings include:

1. Ultrasound screening in suspected fractures may improve X-ray selection and specificity by integrating ultrasound findings into clinical decision rules like the Ottawa Ankle and Knee Rules—limiting X-ray use to patients with positive ultrasound signs of fracture.
2. Evaluation of patients with suspected occult or stress fractures: Ultrasound offers a radiation-free method for early detection, especially when X-rays are negative or inconclusive but clinical suspicion remains high.

Indications specific to hospital-based emergency departments include:

1. Screening for fractures and hematoma during the primary survey in Advanced Trauma Life Support (ATLS), particularly in polytrauma patients, ultrasound enables rapid identification of fractures that are associated with life-threatening hemorrhage (e.g., pelvic or femoral fractures).
2. Detection of instability signs in pelvic and spinal fractures: this is an emerging application where ultrasound can be used to assess dynamic instability or progressive displacement of fractures.
3. Rapid exclusion of fractures in shoulder dislocations: ultrasound can be used prior to reduction to quickly rule out associated fractures, facilitating timely and safe management.
4. Assessment of bone alignment during and after fracture reduction: ultrasound allows immediate verification of anatomical positioning to confirm adequate reduction.
5. Guidance of nerve blocks for pain management and regional anesthesia: ultrasound guidance improves the precision of nerve blocks, which are essential for pain control and safe repositioning of fractures.

## 4. Sonographic Signs of Normal Osseous Anatomy and Fractures

### 4.1. Basic Structure of Bones and Normal Anatomy

The human body contains 206 bones, which are categorized as long, short, flat, or irregular, depending on their shape. Long bones, such as femur and humerus, are longer than they are wide. Short bones, such as those in the wrist, have nearly equal length, thickness, and width. Flat bones, like ribs, are thin and curved. Irregular bones, such as facial bones or vertebrae, do not fit a specific shape. Sesamoid bones, such as the patella, are located within tendons and are not included in the 206 count.

**Figure 1:**
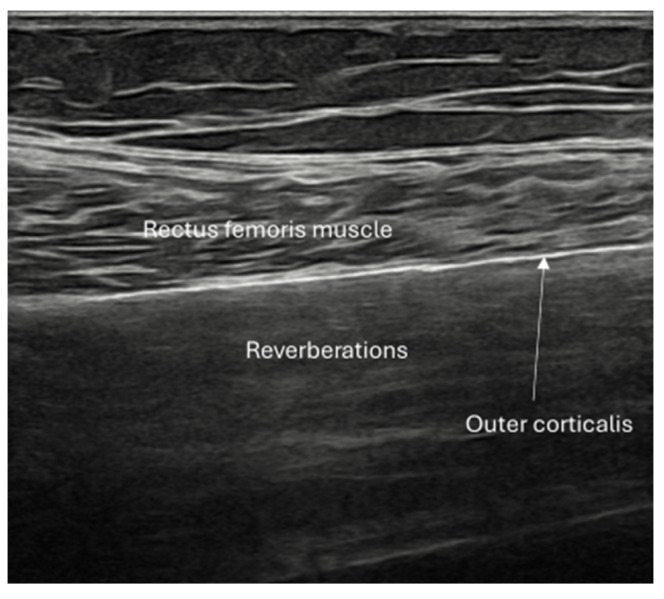
Longitudinal femur. Rectus femoris muscle, reverberations and outer corticalis (arrow) are also indicated.

**Figure 2:**
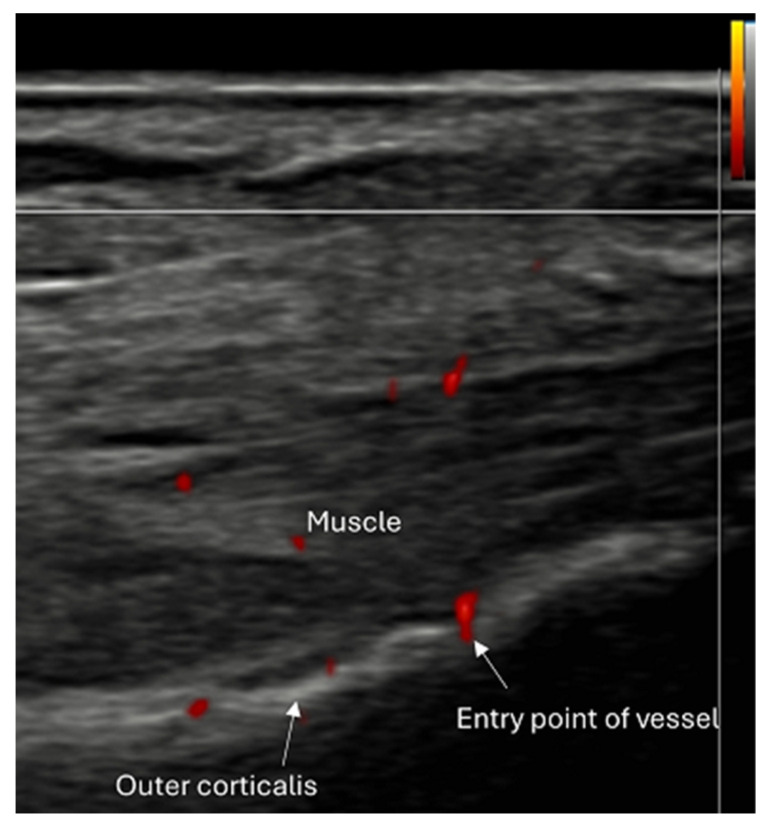
Longitudinal femur. Rectus femoris muscle (muscle), entry points of vessels into the corticalis and the outer corticalis (arrows) are also indicated.

**Figure 3:**
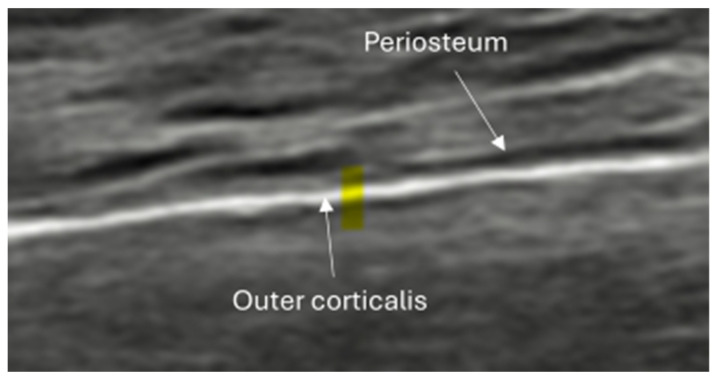
Longitudinal femur. Periosteum and outer corticalis are indicated.

**Figure 4:**
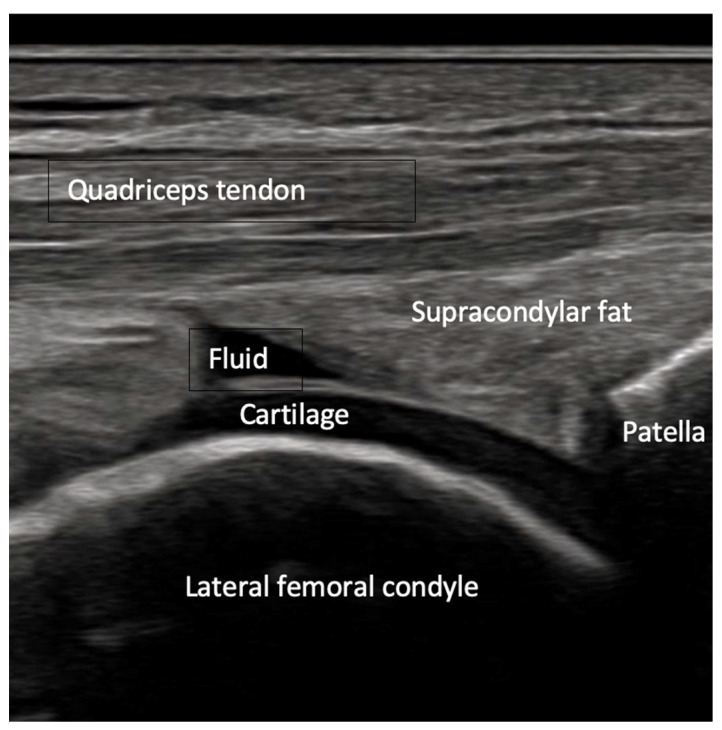
Lateral femoral condyle. Cartilage, patella, supracondylar fat and quadriceps tendon are also indicated. Physiological intra-articular fluid is shown.

Bone is composed of two tissue types: the outer compact (cortical) bone layer and the inner spongy (trabecular or cancellous) bone. When evaluated using ultrasound, the high acoustic impedance difference between the cortical layer and adjacent soft tissues causes near total reflection of ultrasound waves, creating a continuous white line on the screen at the bone–soft tissue interface. This white line can vary in thickness depending on the probe’s angle and the gain setting [Figure 1]. Irregularities or interruptions in this line, such as entry points for vessels, can be seen [Figure 2]. The inner trabecular bone cannot be depicted by typical medical ultrasound waves, which results in anechoic areas or reverberation artifacts. High-resolution probes may differentiate the periosteum as a hypoechoic line above the cortical layer [Figure 3]. Hyaline cartilage, commonly found on articular surfaces, appears as an echo-poor or echoless band, with thickness varying from 1 to 3 mm depending on the bone [Figure 4].

Finally, a phenomenon known as “pseudo-usur” (false-positive erosion of the cortical layer) should be noted. This phenomenon occurs when ultrasound waves strike the cortical layer perpendicularly and are not reflected, resulting in the appearance of a cortical defect. A slight tilting of the probe can correct this and reveal the cortical layer.

### 4.2. Sonoanatomy of Fractures [Figure 5, Figure 6, Figure 7 and Figure 8]

Fractures can be identified on ultrasound using both direct and indirect sonographic signs.

Direct sonographic signs refer to visible disruptions in the bone structure, particularly the cortical layer. These include:

- Irregularity, interruption, or gaps in the cortical line: the cortical bone normally appears as a continuous, bright echogenic line, which is interrupted or irregular in the presence of a fracture.
- Reverberation artifacts within or adjacent to the fracture gap (also known as the “chimney sign”): these repetitive echoes are caused by ultrasound waves reflecting off the fracture surfaces.
- Bulging or abnormal angulation of the cortical layer: deformities or outpouchings of the normally straight cortical surface indicate displacement or bending at the fracture site.
- Dislocation: misalignment of bone fragments, visible as separation or shift from their normal anatomical position.
- Angulation: an abnormal angle formed between fracture fragments, indicating malalignment.
- Osseous avulsions and small bone fragments: detached bone pieces that appear as discrete, hyperechoic fragments adjacent to the main bone.

Indirect sonographic signs suggest the presence of a fracture through secondary changes in the surrounding soft tissues, including:

- Local hematoma or soft tissue edema: fluid collections or increased echogenicity near the fracture site indicating bleeding and inflammation.
- Periosteal thickening or elevation: the periosteum may appear thickened or lifted due to injury or early callus formation.
- Joint effusion and liphemarthrosis: fluid accumulation within a joint, sometimes containing fat droplets, which often indicates an intra-articular fracture.

Occult and stress fractures:

They may present with subtle cortical interruptions accompanied by surrounding soft tissue edema and thickening of the cortical and periosteal layers. Early callus formation may also be visible. Anecdotal evidence suggests that in very subtle stress fractures, reverberation artifacts beneath the cortex may be absent, which can make diagnosis more challenging.

Furthermore, color and power Doppler ultrasound can reveal increased vascularity in the periosteum and surrounding soft tissues [3], indicating active inflammation and healing processes.

By combining these direct and indirect sonographic features, ultrasound can provide a comprehensive evaluation of fractures, including those that are difficult to detect with conventional imaging techniques.

## 5. Examination Technique

Although bone ultrasound is a relatively straightforward imaging modality, it requires a standardized and systematic approach to ensure accurate and reliable results. Adhering to established guidelines is essential to optimize image quality and avoid diagnostic errors. The following recommendations should be considered:

- Use of a silicon pad, generous amounts of ultrasound gel, or a water bath: These methods help minimize patient discomfort caused by transducer pressure on sensitive or injured areas. Additionally, they improve acoustic coupling between the transducer and the skin, which is critical for obtaining high-quality images and reducing artifacts.
- Selection of the appropriate transducer: It is advisable to use the highest possible frequency probe to maximize spatial resolution, particularly when imaging superficial bones. Depending on the clinical application and anatomical region, specialized probes such as linear-convex arrays or small footprint probes (e.g., hockey stick probes) may be preferred to facilitate access to difficult-to-reach areas and improve visualization.
- Adjustment of dynamic range: setting the dynamic range lower than usual enhances contrast resolution, making subtle cortical irregularities and fracture lines more conspicuous on the ultrasound image.
- Focusing and scanning technique: The focal zone should be positioned at the level of the cortical bone to maximize image sharpness. The probe should be oriented perpendicular to the cortical surface and scanned systematically in longitudinal and transverse planes. Scanning in multiple planes—typically three to four different angles—is recommended to avoid missing fracture lines or misinterpreting artifacts.
- Image magnification: excessive zooming or magnification of the image section should be avoided, as this can exaggerate normal cortical irregularities or artifacts, potentially leading to false-positive interpretations.
- Comparison with the contralateral side: whenever possible, imaging the corresponding site on the asymptomatic or unaffected side provides a valuable reference for normal anatomy and helps differentiate pathological findings from anatomical variants.
- Use of color and power Doppler: When employing Doppler techniques to assess vascularity related to inflammation or healing, care must be taken to avoid excessive probe pressure. Over compression of the tissue can collapse small vessels, leading to false-negative findings.
- Role of advanced ultrasound techniques: Currently, there is limited evidence supporting the routine use of elastography or contrast-enhanced ultrasound (CEUS) in fracture diagnosis. Although these modalities offer potential advantages in tissue characterization and perfusion assessment, their clinical utility in fracture imaging remains investigational and is not yet established.

By following these guidelines and maintaining a standardized protocol, practitioners can improve the diagnostic accuracy of bone ultrasound, reduce operator variability, and enhance patient comfort during the examination.

## 6. Systematic Workflow of Ultrasound Application in Fracture Diagnostics and Regional Pain Therapy

The use of ultrasound in fracture diagnostics follows a clearly structured process that enables rapid, targeted, and patient-centered assessment. The process begins with the identification of clinical indications—typically pain, swelling, or trauma—and continues with a systematic examination technique to evaluate the bone status sonographically.

The focus is primarily on visualizing the cortical bone, detecting fracture lines, and assessing associated findings such as hematomas or joint effusions. In addition to diagnostics, ultrasound also allows for the targeted administration of analgesia through nerve or fascial plane blocks directly at the site of injury.

**Chart 1:**
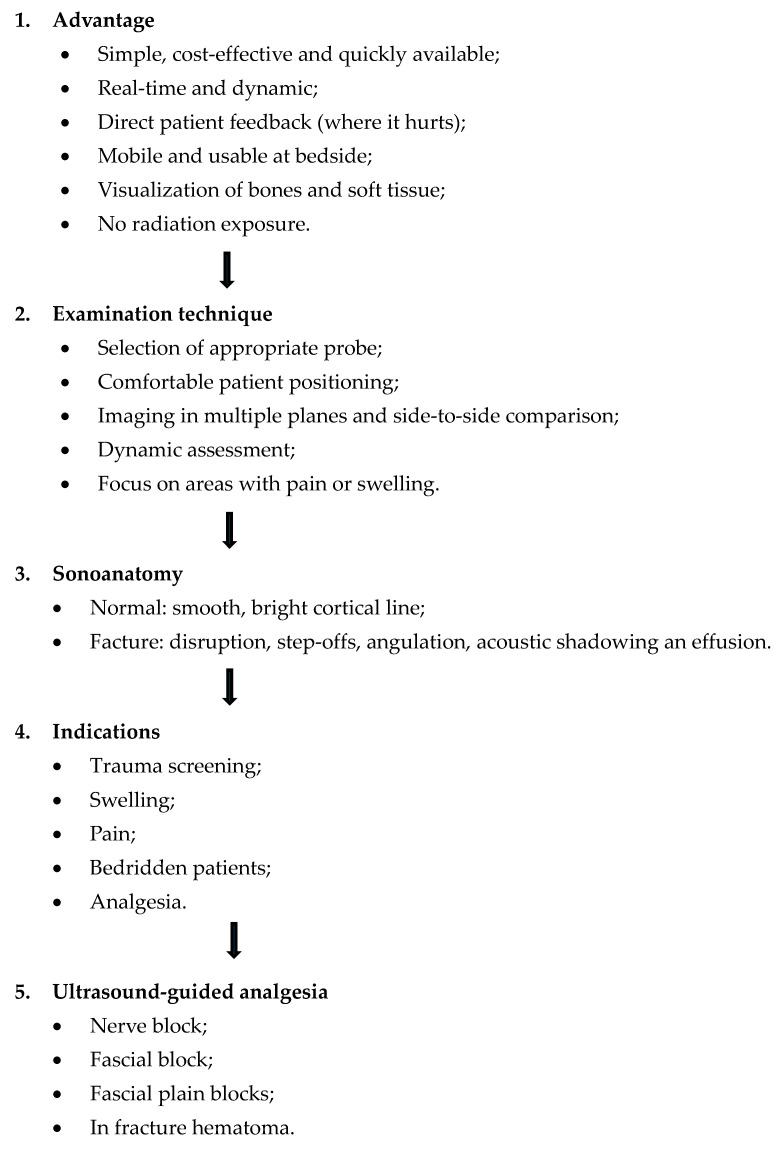
Process Flow: ultrasound in fractures.

The following process flow outlines each step from indication to potential therapeutic application and serves as a practical guide for the effective use of ultrasound in suspected fractures (Chart 1).

## 7. Limitations and Future Directions

- Limitations

Although ultrasound offers several advantages (see Chart 1), it also has notable limitations. The following points summarize the most important drawbacks of this method:

1. Not suitable for all types of fractures: intra-articular fractures often need complimentary imaging: X-ray and/or CT.
2. Limited access to deep or anatomically complex fractures: anatomical regions such as hip or spine are difficult to evaluate using ultrasound.
3. Intrinsic limitations due to bone structure: Only the bone surface is visible. The internal architecture remains inaccessible.
4. Operator dependency: detecting fractures of small bones and small fractures require considerable expertise.
5. Lack of standardization: there are few standardized protocols for ultrasound-based fracture assessment in routine clinical practice.

- Future directions

Emerging and future directions in fracture ultrasound with potential clinical impact include:

1. Fracture healing monitoring: Ultrasound facilitates early callus formation. This can be useful for radiation-free monitoring of healing progression.
2. Portable use in prehospital medicine: handheld ultrasound devices permit rapid fracture assessment at the site of injury, including sports, military and emergency rescue service environment.
3. Integration of artificial intelligence for image analysis: machine learning algorithms can assist in the automated identification of bone discontinuities or callus formation, thereby enhancing diagnostic accuracy.
4. Dynamic real-time imaging during movement: Ultrasound allows for functional assessment under motion. This is helpful for detecting joint instability and diagnosing complications such as pseudarthroses.
5. Multimodal imaging fusion (e.g., MRI-ultrasound fusion): combining ultrasound with other imaging techniques can augment diagnostic capabilities, especially for complex fractures and for assessing surrounding soft tissue injuries.

## 8. Narrative and Illustrative Review

Existing evidence shows that fracture ultrasound is a valuable diagnostic bedside tool [1]. In this section, the state of the art of seven indication groups is summarized and illustrated with examples.

### 8.1. Screening of Patients with Suspicion of Fracture to Improve the Specificity of X-Rays [Figure 9 and Figure 10]

The indications for conventional X-rays to diagnose fractures are often based on subjective criteria, with clinical examination and forensic considerations taking priority. This approach leads to many unnecessary radiographs, as demonstrated by studies on ankle fractures [4]. As a result, rules were developed to more precisely determine when X-rays are indicated, though these rules apply only to a few anatomical regions. Meta-analyses on fractures of the knee, ankle, and foot have shown improved diagnostic efficiency [5,6,7]. Although these studies report high rule-out rates for fractures, their rule-in rates, with specificities below 50%, remain unsatisfactory. We believe that ultrasound as a first-line imaging tool can reduce unnecessary X-rays by reliably ruling out fractures. Additionally, ultrasound is highly beneficial in cases where no standardized rules are available. If ultrasound rules out a fracture, X-rays can be omitted. This hypothesis was confirmed by a study conducted by Hedelin et al. [8]. Junior orthopedic surgeons were trained for 30 min to rule out ankle fractures, and in 122 patients with a positive Ottawa Ankle Rule indicating X-ray, 85 (70%) X-rays could have been avoided. In addition, patients with ankle injuries were treated more quickly, which reduced treatment costs, length of stay, and radiation exposure. However, to our knowledge, there is a lack of studies confirming these findings for other fractures.

Recommendation 1: Ultrasound should be used as a screening tool for suspected fractures, followed by further imaging (X-ray or CT) if necessary. In cases of obvious simple fractures, conventional X-ray remains the preferred method.

### 8.2. Screening of Patients with Suspicion of Occult and Stress Fractures (Figure 11 and Figure 12)

**Figure 11:**
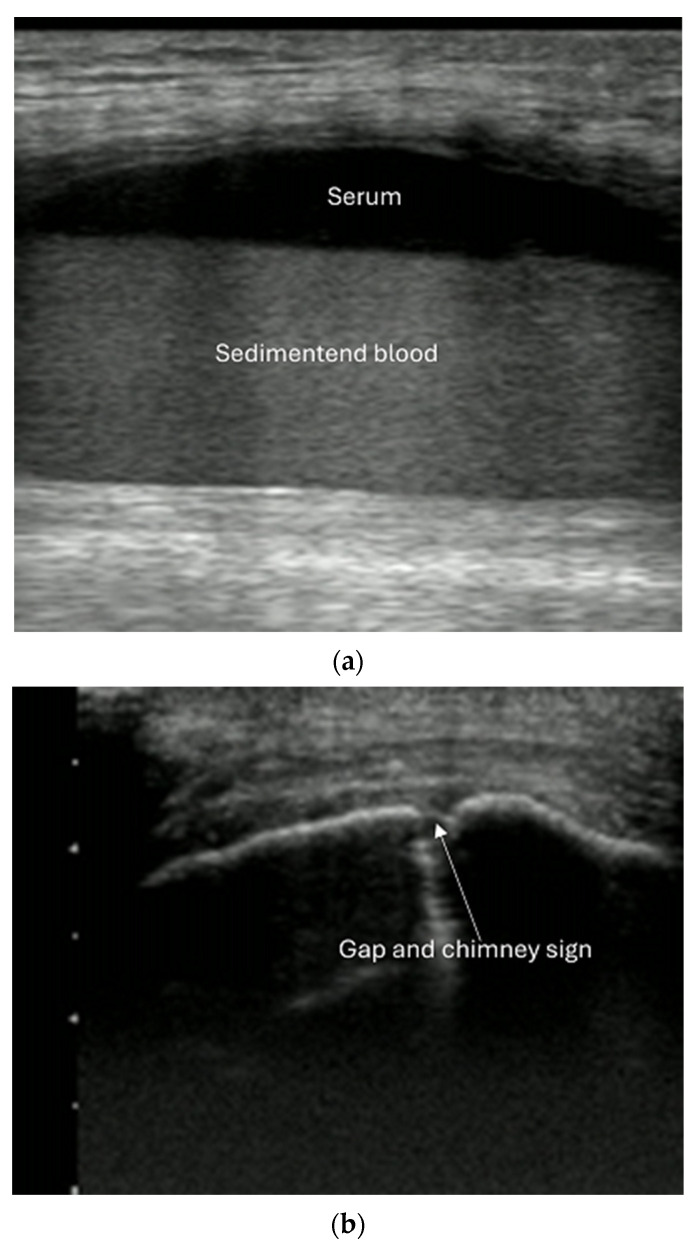
Occult fracture with negative X-ray. (**a**) Hemarthrosis of lateral knee recessus. (**b**) Lateral femoral condyle with gap and chimney sign.

Ultrasound use in occult fracture [Figure 11].

**Figure 12:**
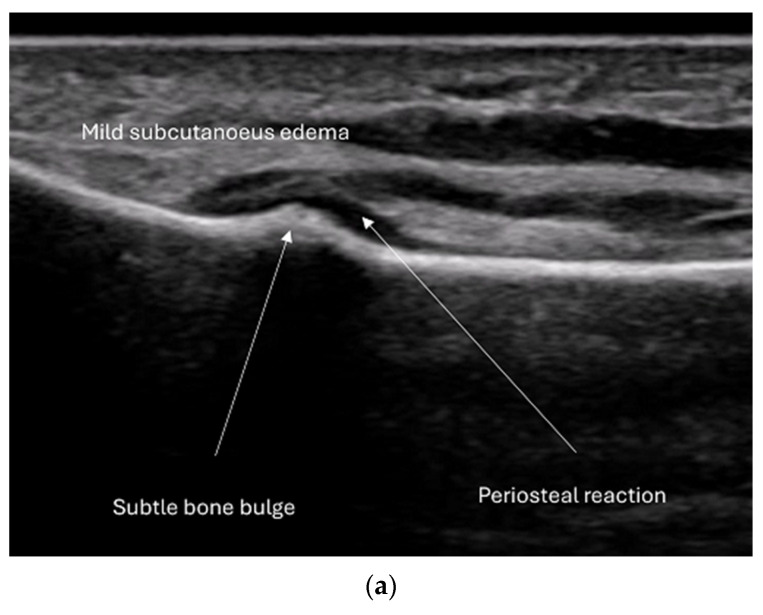

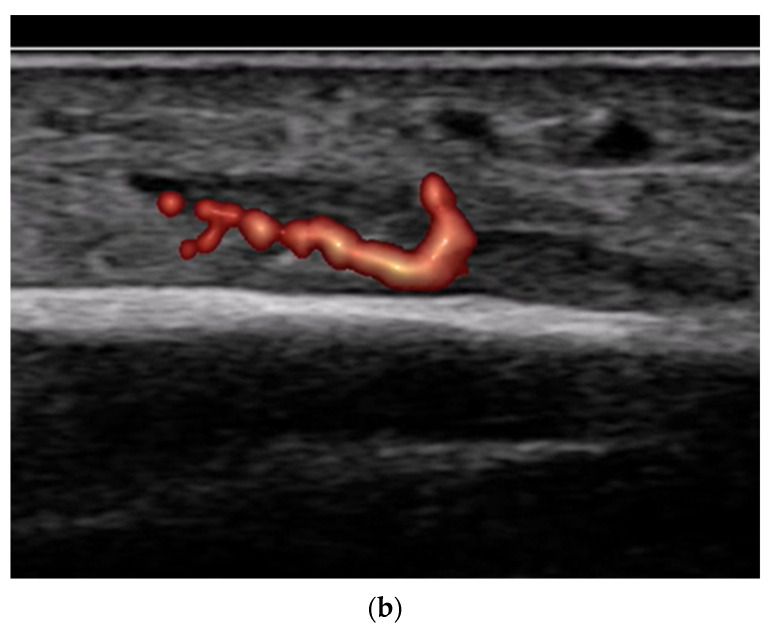
Stress fracture metatarsal. (**a**) Subtle bone bulge, periosteal reaction and mild subcutaneous edema. (**b**) Same patient with power Doppler shows inflammatory vessels.

CT is typically used to diagnose suspected radio-occult fractures in acute settings, whereas MRI is employed when early diagnosis or soft tissue evaluation is needed. Bone scintigraphy or PET/CT does not play a role in the emergency setting. Although a comprehensive evaluation of ultrasound’s role in this area is still lacking, early evidence suggests that ultrasound could be used more frequently for suspected occult fractures, given its increasing availability and ease of use. It is recommended as first-line imaging, except for suspected spine and pelvis fractures, where ultrasound imaging is inadequate.

Acute, symptomatic occult bone fractures often remain undetectable on initial X-rays, becoming visible only days later when a callus forms. A recent meta-analysis demonstrated the superiority of ultrasound over conventional radiology for detecting radio-occult sternal and rib fractures [9], and ultrasound can even identify cartilage rib fractures [10]. While these fractures are typically not problematic, very painful dislocations can occur and may require surgical intervention. This diagnosis is often missed on radiography.

Although there is limited evidence, studies have shown that ultrasound can detect occult fractures in the upper and lower extremities. Examples include fractures of the humeral greater tuberosity, radial head, scaphoid, hamate, trapezium, and sesamoid bones in the upper extremity, and femoral head, patella, tibia, malleoli, talus, calcaneus, cuboid, and fifth metatarsal in the lower extremity [11,12,13,14,15].

Intra-articular fractures are missed on X-ray in up to 15% of cases [16] and carry a high risk of later complications if left untreated. These fractures can cause joint effusion (hemarthrosis) and are associated with “fat pad signs” on X-rays in joints such as the elbow, knee, and ankle. However, ultrasound provides a reliable means of visualizing joint fluid across all joints. The fluid appears anechoic (or hyperechoic) dependent on the stage of hemorrhage) and can be displaced by pressure. Although direct visualization of intra-articular fractures is often not possible with ultrasound, the technique has shown promise for detecting effusion and related fractures.

A large case series [17] demonstrated that ultrasound could detect joint effusions in patients with a positive fat pad sign who had no direct evidence of a radial head or neck fracture on X-ray. Fractures missed on X-ray were identified with ultrasound, supporting the role of ultrasound in detecting radio occult radial head and neck fractures.

Another specific sign of intra-articular fractures is liphemarthrosis. This phenomenon occurs when bone marrow fat and blood leak into the joint, with the fat floating on top of the blood. Ultrasound reveals bright spots (fat droplets) in the dark effusion (blood), and when immobilized, the effusion forms two layers (fat on top, blood underneath). Studies have shown that ultrasound has high sensitivity and specificity for identifying liphemarthrosis in knee fractures [16].

Recommendation 2: Given the promising but limited evidence, ultrasound should be considered a first-line imaging tool for suspected occult fractures, except in the pelvis and vertebral column.

Ultrasound use in stress fractures [Figure 12].

Stress fractures, which are small cracks in the bone, can result from either repetitive loading of weak bone (fatigue fractures, FF) or overloading of normal bone (insufficiency fractures, IF) [10]. FFs are common in older individuals and often affect the pelvis, femoral neck, knee, calcaneus, and metatarsals. IFs typically occur in athletes, particularly in the lower extremities. Ultrasound can detect changes in the soft tissue, periosteum, and cortical bone of stress fractures. Early changes are subtle and include poorly demarcated soft tissue edema and periosteal thickening. Inflammatory changes can be observed on color Doppler imaging. If a stress fracture is detected, further work-up with X-ray is necessary to rule out pathological fractures.

Recommendation 3: Ultrasound should be used as first-line imaging for suspected stress fractures, with follow-up X-ray if evidence of a stress fracture is identified. Exceptions include pelvic and vertebral column fractures.

Hospital-based Emergency Departments.

1. Screening of fractures in the primary ATLS (Advanced Trauma Life Support) survey that are associated with or may cause life-threatening bleeding in polytrauma

Fracture ultrasound is seldom mentioned in the context of major trauma management; however, its potential role should not be underestimated. For example, open-book fractures of the bony pelvis, which are often associated with vascular injuries and retroperitoneal hematomas, can lead to severe hemorrhagic shock. Early diagnosis and hemostasis can significantly reduce morbidity and mortality [18].

Ultrasound of the symphysis, integrated into E-FAST (Extended Focused Assessment with Sonography for Trauma), can play an important role in diagnosing these fractures quickly, even in prehospital settings. Iannello et al. [19] reported that a symphysis gap wider than 2.5 cm is correlated with open-book fractures. Ultrasound can be used at the accident site to verify pelvic fracture positioning.

In shock patients without evidence of bleeding in the torso, ultrasound can quickly identify extremity fractures and hematomas, although studies on this topic are still lacking.

Recommendation 4: The E-FAST protocol, indicated for severely injured patients in shock, should be expanded to include fracture screening of long tubular bones and open-book fractures when the torso scan is negative for free fluid. This could identify hidden injuries and bleeding.

2. Screening for instability in pelvic and spine fractures

Pelvic fractures are often associated with instability, which requires surgical intervention to prevent bleeding and facilitate early mobilization. Testing the stability of pelvic fractures is challenging, but dynamic ultrasound has shown promise as a potential diagnostic tool [20,21]. The gold standard for diagnosing vertebral fractures is computed tomography (CT), but it is costly, involves high radiation exposure, and may not be available in all settings. A study in 2021 on cervical spine injuries showed that ultrasound could detect fractures, but its high rate of missed fractures limits its routine use for excluding spinal fractures [22]. However, other studies [23] have highlighted ultrasound’s potential for detecting unstable cervical spine fractures.

The diagnosis of thoracolumbar spine fractures, particularly ruptures of the posterior ligamentous complex (PLC), is best achieved through MRI, but when MRI is unavailable, ultrasound can serve as a useful alternative. A meta-analysis found that ultrasound had 89% sensitivity and 100% specificity for detecting PLC ruptures [24].

Recommendation 5: In settings where CT or MRI is unavailable, ultrasound should be used during the secondary survey to help identify signs of instability in spinal injuries.

**Figure 13:**
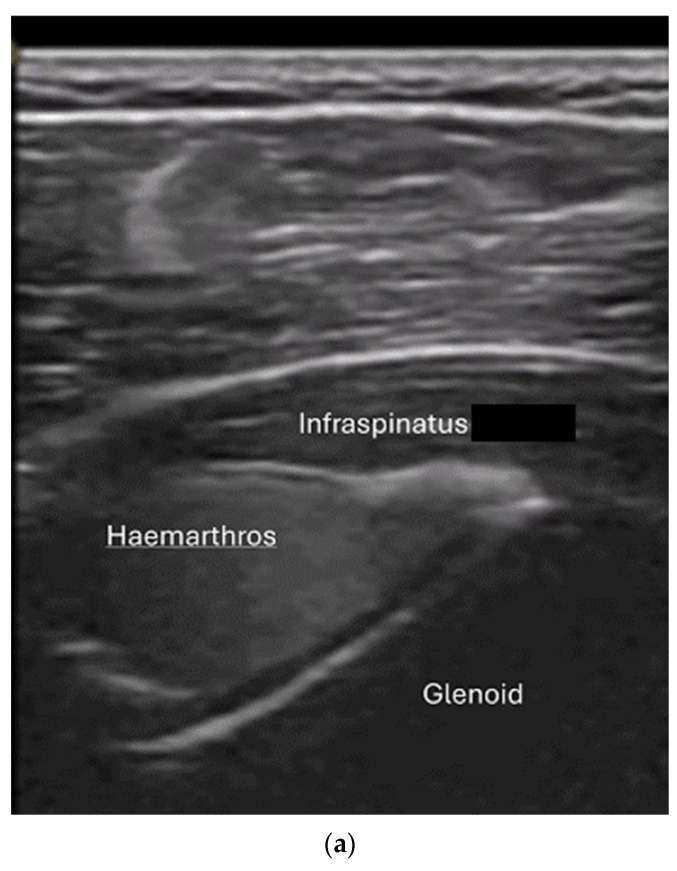

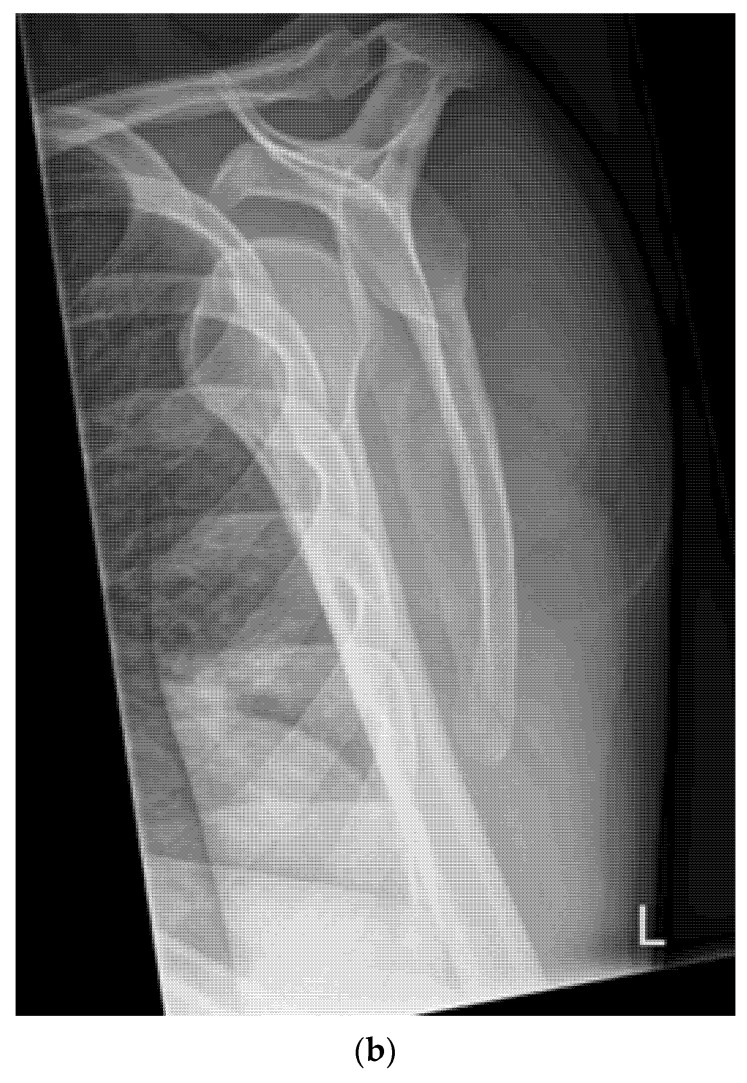
Anterior dislocation of left shoulder. (**a**) Ultrasound posterior transversal view. (**b**) X-ray of the same patient.

3. Quick exclusion of fractures in shoulder dislocations for timely reduction (Figure 13).

Shoulder dislocations are extremely painful for those who are affected. Reduction in patients with a significant trauma mechanism or those who aged > 40 years should not be performed without radiography to rule out significant fractures. On the other hand, if a reduction maneuver is performed on a patient without shoulder dislocation but with certain fractures, the maneuver is very painful and can lead to significant fracture dislocation. In addition, shoulder dislocations with certain fractures of the humeral head or glenoid can be an indication for open reduction. Therefore, reductions outside an emergency or other ambulatory medical facility equipped with X-ray, or a hospital are often not possible. In addition, waiting times for radiographs can be considerable in many health institutions. Because even high-dose opioids frequently offer inadequate pain relief or cause unwanted side effects, patients can suffer considerably while awaiting diagnosis and management, not only during transportation to the hospital but also during waiting times and transport to imaging. The potential side effects of pain medication also mean that patients must be monitored for a longer period after successful repositioning, placing further strain on the infrastructure and personnel resources.

In this situation, PoCUS is a highly welcome, quickly available bedside imaging tool with a sensitivity and specificity shown to be 100%, including the repository result in a meta-analysis of 1836 patients and 636 dislocations [25]. A new prospective randomized controlled study conducted in 2022 with 1206 patients with shoulder injuries showed that ultrasound was highly accurate and superior to physical examination alone, could rule out significant fractures, and was a highly accurate diagnostic tool for dislocations and relocation diagnosis [26]. The fringe benefits of PoCUS use in suspected shoulder dislocations are considerable: avoidance of unnecessary transportation and waiting times to exclude a fracture and determine if there is a need for a closed reduction. Patients can be spared prolonged severe pain, and, as a consequence, the probability of a successful reduction with little or no analgesia can be increased. The length of stay and staff utilization can be greatly reduced. Finally, PoCUS could be used to treat many patients directly at the scene of the accident or at the primary care practice.

Recommendation 6: Ultrasound should be integrated into the assessment and management of shoulder injuries, including proximal humerus fractures, shoulder dislocations, and post-relocation maneuvers, both prehospitally, in emergency departments, and other acute care settings.

4 Evaluating bone position during and after reduction to avoid considerable radiation exposure (video)

Checking the position of fractures is all about axial deviation and stability. An X-ray image intensifier is often used for this purpose. However, this can be easily determined by ultrasound. Several planes are required for accurate assessment. In contrast to children, the use of ultrasound for fracture reduction in adults is still in its infancy. The advantages are obvious: no expensive infrastructure, no radiation exposure, faster execution, improved precision thanks to real-time imaging, and reduced risk of injuring nerves and blood vessels.

We could only find a few studies on this topic, mainly limited to the distal radius. In a systematic review, the reduction site was correctly demonstrated, and the quality of the reduction was improved compared with the standard procedure. However, no conclusions could be drawn regarding the benefits for patients [27]. A pilot study involving five patients (radius, metacarpus, and fingers) revealed a potential role for wider application of ultrasound in fracture reduction in the emergency room [28].

Another application mentioned in the literature is closed reduction of nasal fractures, which is often performed under general anesthesia. Kim DH et al. presented a case in which ultrasound in the operating room was used to avoid unnecessary manipulation, thereby reducing the nose under local anesthesia [29]. We wonder whether this technique could be applied in the emergency department and save considerable resources, costs, and time and are awaiting the results of such a study. However, conventional linear probes cannot be satisfactorily coupled to the nose. Silicone pads could help in such situations. Shigemura et al. suggested using water as a contact medium for ideal imaging and present a special method, which appears to us somewhat impractical [30].

Recommendation 7: The potential of ultrasound for positional control of fracture during fracture reduction in emergency situations should be explored in emergency departments.

5 Nerve, fascia and fascial plane blocks

**Figure 14:**
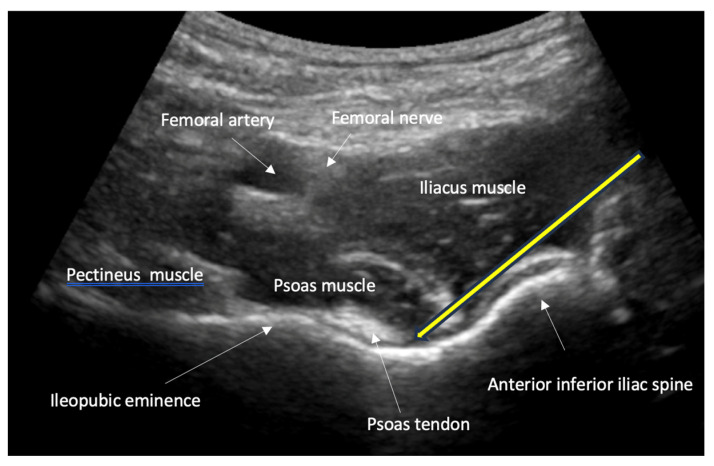
Extended view of the inguinal region for the PENG block (yellow arrow). Femoral artery, femoral nerve, iliacus, psoas and pectineus muscles as well as ileopubic eminence, psoas tendon and anterior inferior iliac spine are also shown.

Nerve, fascia, and fascial plane blocks are currently the evidence-based standard for certain pain treatments and minor procedures in emergency patients. Fractures of the ribs, hip, and femoral neck are extremely painful. Analgesia in these situations has a very high priority, and even with high-dose opioids, it is not always satisfactory, in contrast to nerve blocks. Ultrasound-assisted nerve blocks are highly precise, effective, safe, and quick to perform. In emergency medicine, they are increasingly being replaced by so-called fascia and fascial plane blocks. The local anesthetic is injected under the fascia near the nerves and flows in this space to and around the nerves. Their advantage over nerve blocks is a simpler technique, protection of the nerves, and wider nerve coverage with a longer-lasting effect. The most common block for femoral head/neck fractures placed by emergency physicians is the fascia iliaca block. Nevertheless, the PENG (pericapsular nerve group) fascial block [Figure 14] appears to provide the best analgesia for femoral neck fractures among the available blocks [31,32]. There are also various options for rib fractures [33]. The optimal technique depends on the individual case and the preference of the treating team. The serratus anterior fascial block is easy to perform and safe [34].

Finally, the reduction of fractures is extremely painful. In emergency departments, conscious or deep sedation is mostly used for this purpose, with the associated risks. Nerve blocks, preferably placed under ultrasound guidance, achieve the same effect but with fewer adverse events and a shorter length of stay in the emergency department [35].

Fracture hematoma blocks are another option for pain control and to reduce required systemic sedation if reduction is required. These blocks are performed immediately pre-reduction with a local anesthetic, such as lidocaine or bupivacaine, and a strict aseptic technique. The medication is injected directly into the fracture hematoma and preserves the motor and sensory functions of the injured area. After the hematoma block has been set, a closed reduction can be performed with minimal discomfort to the patient. However, it is of the utmost importance to utilize aseptic techniques to avoid infection.

Recommendation 8: Simple fascia and fascial blocks are highly suited for analgesia in patients with rib and femoral neck fractures. Local anesthesia using nerve blocks and fracture hematoma blocks should be preferred over analog sedation for fracture reduction whenever possible, as they are less risky.

## 9. Clinical Case Examples—Advantages and Limitations of Ultrasound Compared to X-Ray and CTC

Based on selected clinical case examples, the respective strengths and weaknesses of ultrasound are presented in direct comparison with conventional imaging modalities such as X-ray and computed tomography (CT). Using medical image examples of identical cases, the diagnostic value of each modality is analyzed across various clinical scenarios. The aim of this comparative approach is to support the selection of the most appropriate imaging technique in clinical practice and to provide a deeper understanding of the capabilities and limitations of ultrasound in comparison to X-ray and CT.

- Example: Hemorrhagic Shock Due to Distal Femur Fracture—Rapid Diagnosis via Ultrasound

A 72-year-old obese female patient was urgently transferred from a rehabilitation facility to the emergency department due to acute right thigh pain during the night, accompanied by signs of hemodynamic shock. The patient denied any fall but reported frequent transient episodes of altered consciousness. Her medical history included a recent subdural hematoma.

**Figure 15:**
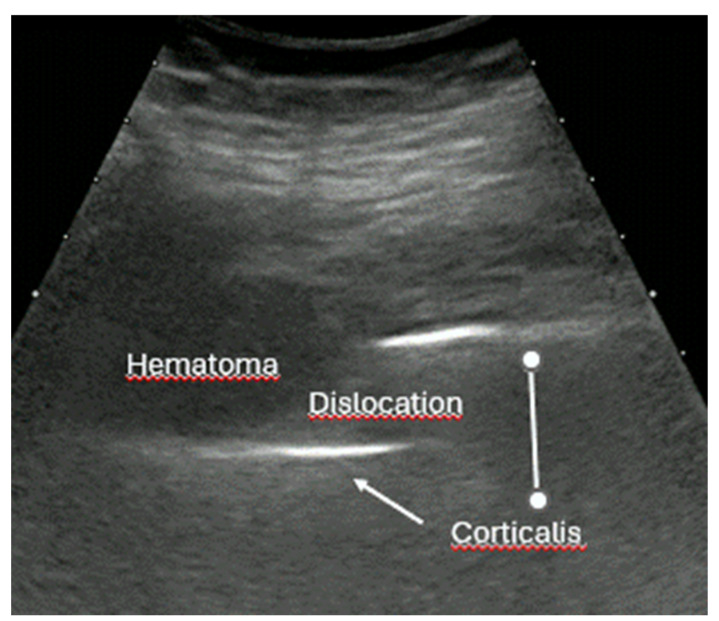
Ultrasound distal femur longitudinal axis with large hematoma and distal fracture.

Initial eFAST was unremarkable. Given the localized pain, focused sonography of the right thigh was performed, revealing a large hematoma and suggesting a distal femur fracture (Figure 15). Anticoagulation therapy was only identified later, supporting the diagnosis of hemorrhagic shock.

**Figure 16:**
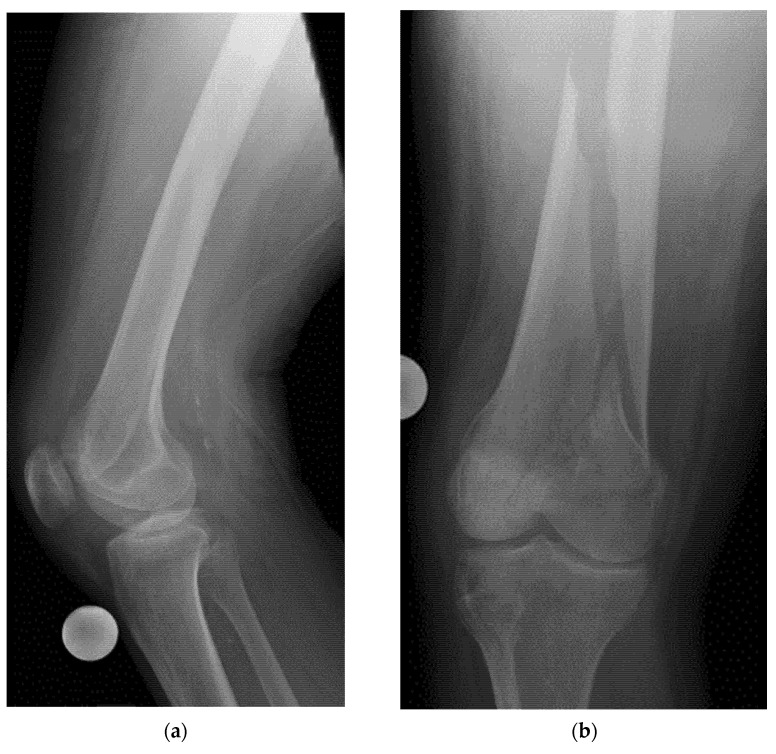
X-ray distal femur fracture. (**a**) Longitudinal axis. (**b**) Transversal axis.

The patient was promptly stabilized with fluids, blood transfusions, and coagulation factors. Conventional radiography subsequently confirmed the distal femur fracture (Figure 16), and after normalization of coagulation parameters, surgical treatment was performed.

Conclusion: In this complex and unclear clinical situation, targeted ultrasound enabled rapid identification of the bleeding source and guided immediate therapeutic measures. While the fracture could not be fully visualized sonographically, the presence of a large hematoma indicated ongoing internal bleeding. Conventional imaging confirmed the diagnosis and allowed for surgical planning. Without bedside ultrasound, critical time might have been lost before reaching the correct diagnosis.

- Example: Stress Fracture

A 63-year-old athletic man experienced a sudden, severe pain below the right lateral knee joint after an intense week of cross-country skiing, without any history of trauma. He was only able to walk with the aid of poles. There was marked tenderness on palpation over the proximal lateral fibula. The physical examination was unremarkable, except for localized tenderness over the proximal fibula and at the insertion of the peroneus brevis muscle, with pain provoked by foot pronation. A diagnosis of insertional tendinopathy was initially suspected.

**Figure 17:**
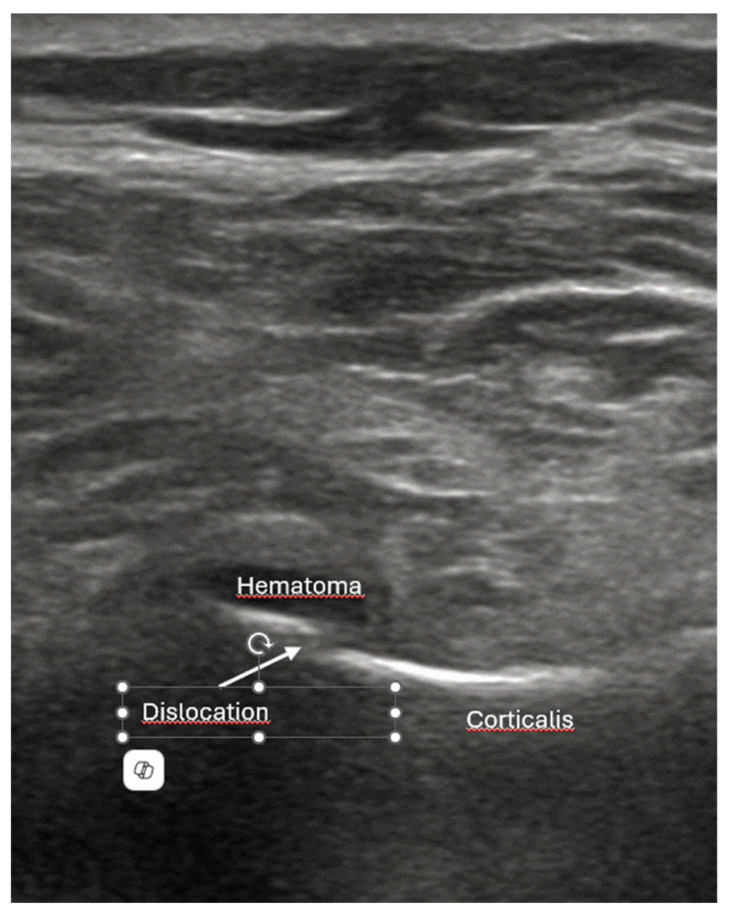
Ultrasound latero-proximal fibula in longitudinal axis.

**Figure 18:**
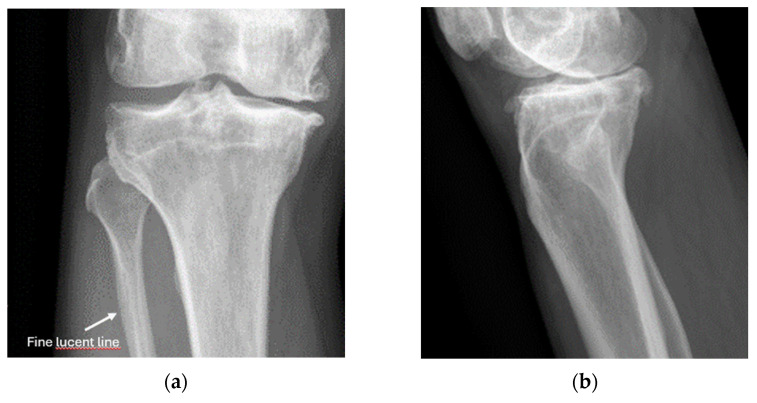
X-ray distal femur fracture. (**a**) Ap view. (**b**) Lateral view.

Ultrasound was used as the first-line imaging modality. It revealed, at the point of maximum tenderness, a subtle but definite disruption in the lateral fibula cortex, accompanied by hypoechoic elevation of the periosteum (Figure 17). To better characterize the suspected stress fracture, a plain X-ray was performed (Figure 18).

Conclusion: In the anteroposterior (AP) X-ray, a very subtle abnormality (a fine lucent line in the lateral cortical bone) is visible. This finding would likely have been missed without the prior knowledge provided by the clear ultrasound result.

- Example: Suspected left shoulder dislocation

A 52-year-old man was admitted to the emergency department after a bicycle accident, presenting with severe pain and a suspected left shoulder dislocation.

**Figure 19:**
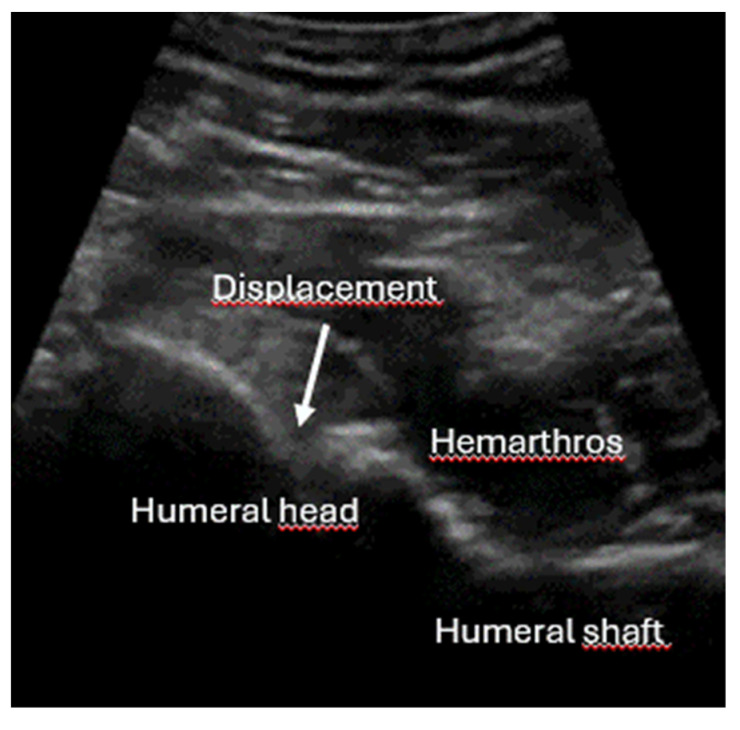
Ultrasound posterior longitudinal view shoulder.

The first-line imaging with ultrasound revealed a multifragmentary, impacted, and medially displaced subcapital humerus fracture (Figure 19). Shoulder reduction was therefore not an option and would have been harmful.

**Figure 20:**
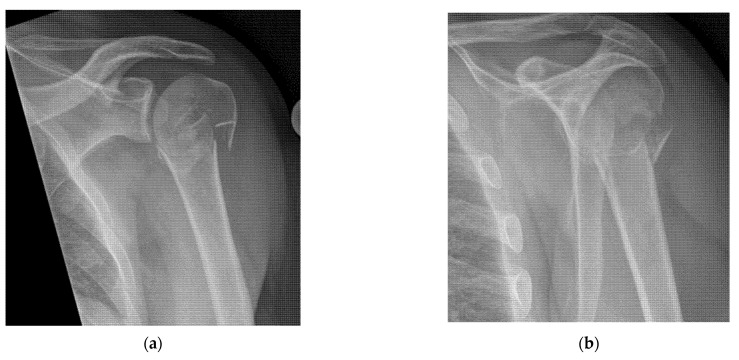
X-ray shoulder with subcapital humeral fracture. (**a**) Shoulder ap. (**b**) Neer view.

Conclusion: Several patients were awaiting X-ray imaging, which was temporarily occupied with a polytrauma case. The suspected diagnosis of “shoulder dislocation” could be ruled out within minutes using ultrasound. As a result, the patient no longer had an urgent indication for immediate radiography and did not need to be prioritized. The delayed follow-up X-ray demonstrated a comminuted subcapital fracture of the humerus (Figure 20).

- Example: Common Case of “occult” rib Fractures

A 65-year-old multimorbid man presented two days after a fall, reporting severe, diffuse pain in the lower left lateral thorax radiating to the back.

**Figure 21:**
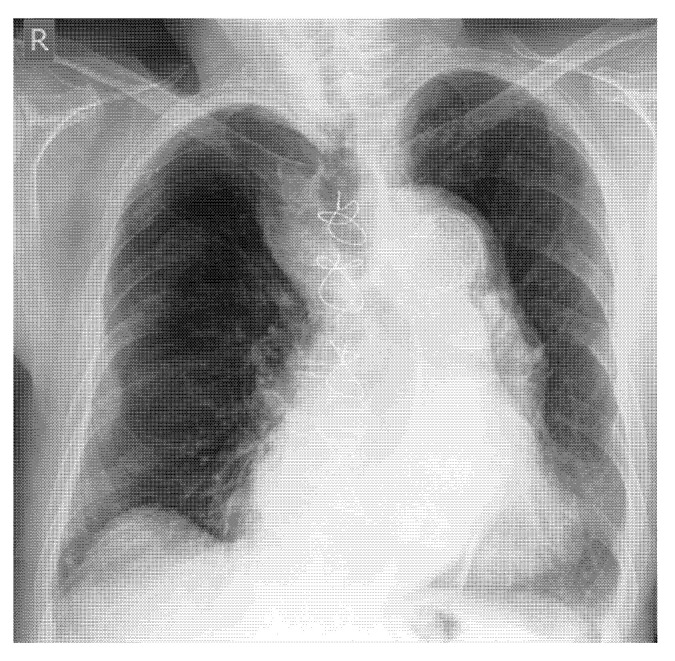
AP chest X-ray with no rib facture.

**Figure 22:**
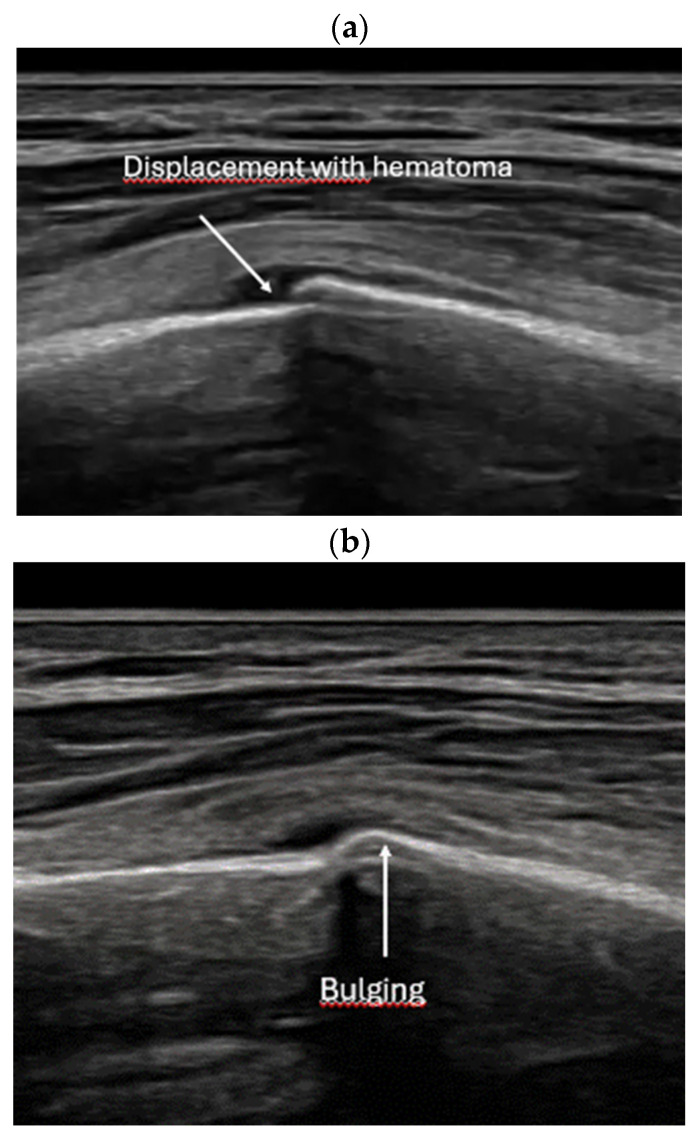
Ultrasound longitudinal rib fractures. Slightly displaced fracture rib (**a**) and non-displaced fracture rib (**b**).

The initial chest X-ray did not reveal any rib fractures (Figure 21). However, ultrasound imaging clearly showed two non-displaced rib fractures (Figure 22a,b).

Conclusion: Several patients were awaiting X-ray imaging, which was temporarily occupied with a polytrauma case. The suspected diagnosis of “shoulder dislocation” could be ruled out within minutes using ultrasound. As a result, the patient no longer had an urgent indication for immediate radiography and did not need to be prioritized.

Example: Occult hip joint fracture

**Figure 23:**
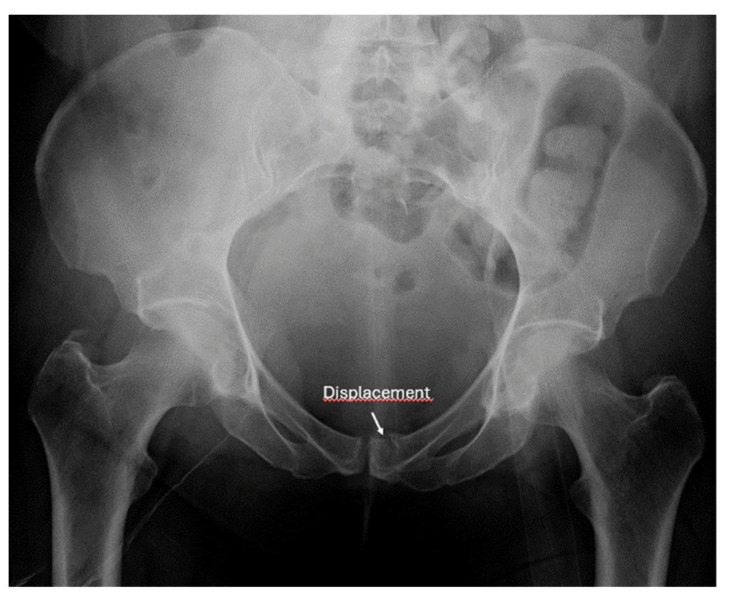
Pelvis X-ray in ap, with left pubic ramus fracture.

**Figure 24:**
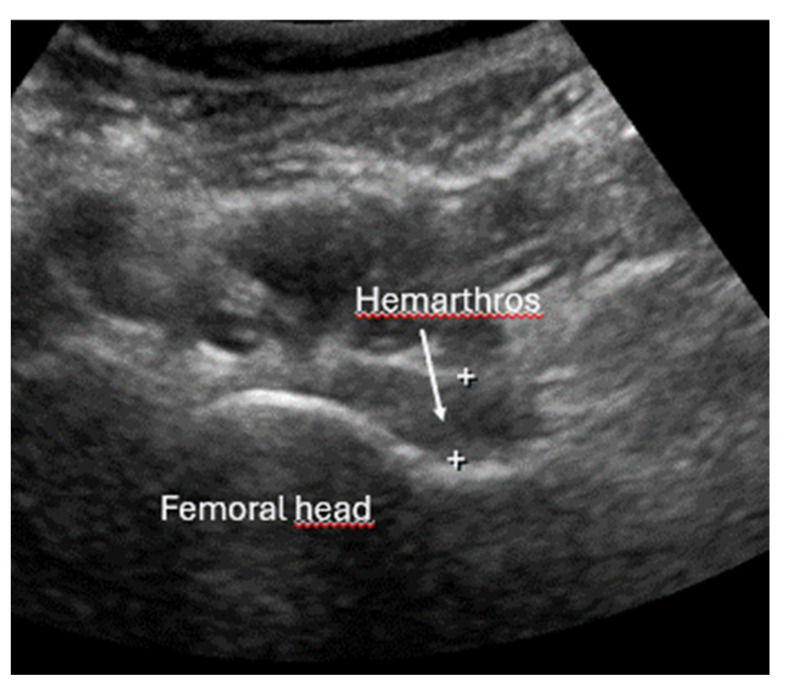
Ultrasound left hip longitudinal view with hemarthrosis.

**Figure 25:**
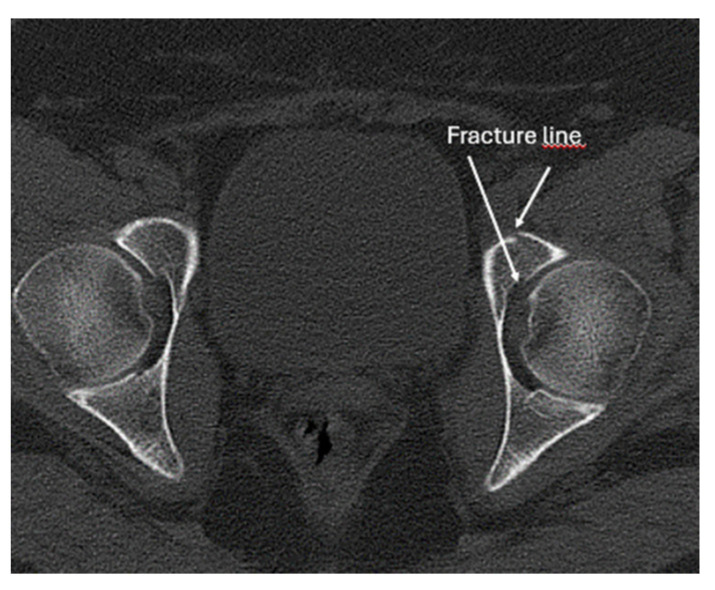
CT scan of the pelvis at the level of the acetabulum with fracture line left.

A 57-year-old male patient was admitted to the emergency department after a fall. He was unable to walk and reported pain in the left hip, particularly with rotational movement. Initial imaging with X-ray revealed a pubic ramus fracture (Figure 23). Due to persistent rotational hip pain, an ultrasound was performed. A large joint effusion was detected (Figure 24), raising suspicion of an occult intra-articular fracture. A CT scan subsequently confirmed the diagnosis of an acetabular fracture (Figure 25).

Conclusion: A plain radiograph with a good overview was the logical first-line imaging modality in this case. It revealed only a pubic ramus fracture. However, based on the clinical examination showing rotational pain of the left hip, an occult hip joint fracture was suspected, justifying the use of ultrasound. The detection of a hemarthrosis provided sufficient indication for a CT scan, which ultimately confirmed the diagnosis.

## 10. Conclusions

- The sonoanatomy of fractures is simple and can be learned quickly. It does not place high demands on the technical equipment of the devices or the skill of the examiner.
- The examination technique is simple. If available, high-frequency linear probes with a right-angled position on the cortical bone and, if necessary, silicone pads for coupling should be used. Important knobology parameters, such as a low dynamic range, non-excessive magnification, and focus on the cortical bone, should be set. The bone should be scanned longitudinally, compared with the healthy side, and not pressed too hard when using the color/power Doppler.
- Seven indications are recommended. Ultrasound should be used:

1. To screen for suspected fracture, and, if positive, follow-up by further imaging (X-ray or CT if indicated).
2. As first-line imaging for suspected occult or stress fractures.
3. In E-FAST, which is indicated for moderately to severely injured patients with shock, it can be expanded to fracture screening of long tubular bones and open-book fractures if the result of the torso scan is negative for free fluid.
4. In the secondary survey to help identify signs of spinal injury instability, whenever CT and MRI are not available.
5. Integrated into the assessment and management of shoulder injuries, including proximal humerus fracture, shoulder dislocation, and post-relocation maneuver, both pre-hospital and in emergency department settings.
6. Positional control of fractures during and after reduction.
7. For simple fascia and fascial blocks, such as rib and femoral neck fractures. Local anesthesia using nerve blocks is preferred to analog sedation for fracture reduction.

- The consistent use of ultrasound for a given indication could save millions of X-ray examinations worldwide.
- This growing body of evidence supports a broader role for ultrasound as a frontline imaging modality in fracture diagnosis.

## Figures and Tables

**Figure 5:**
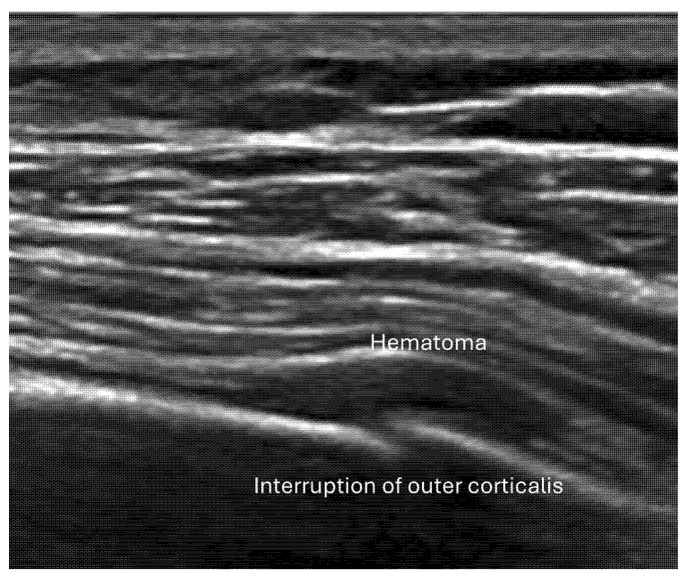
Non-dislocated rib fracture. Interruption of outer corticalis and hematoma are indicated.

**Figure 6:**
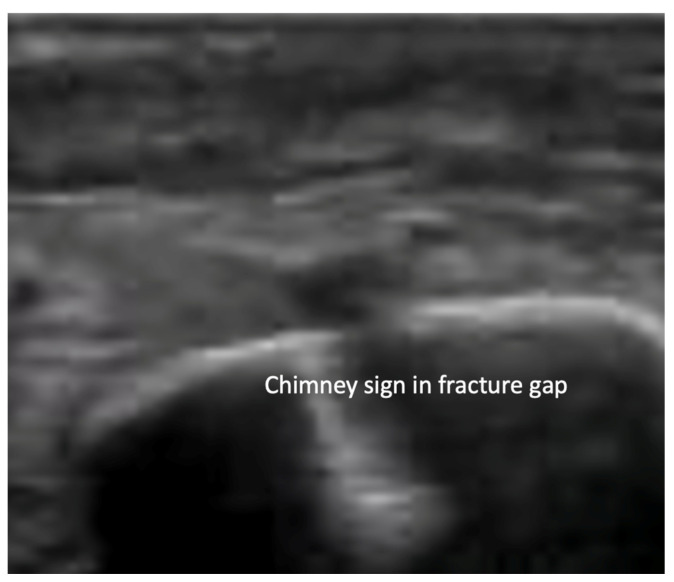
Non-dislocated lateral ankle fracture. The chimney sign in the fracture gap is indicated.

**Figure 7:**
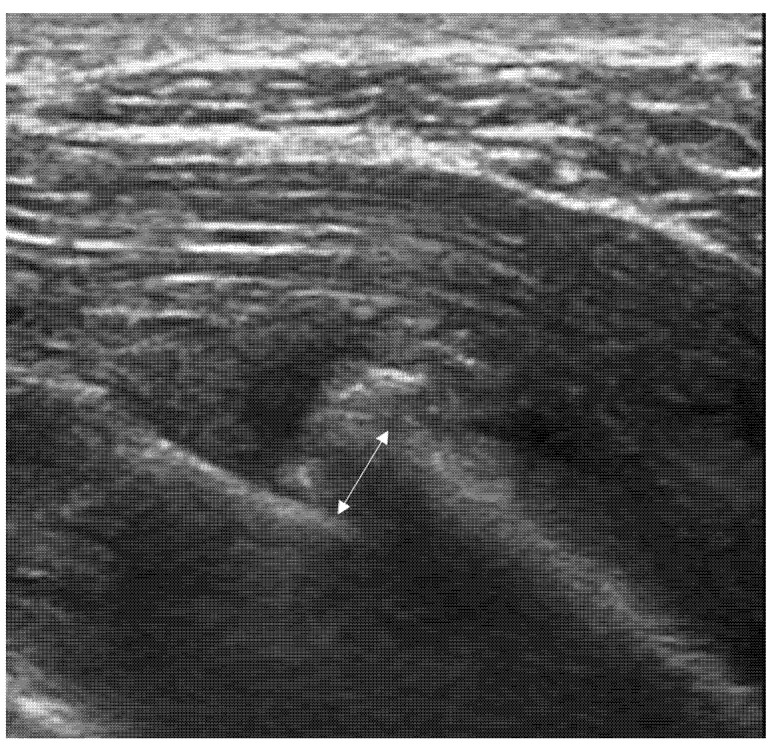
Dislocated rib fracture. Dislocation is shown by arrows.

**Figure 8:**
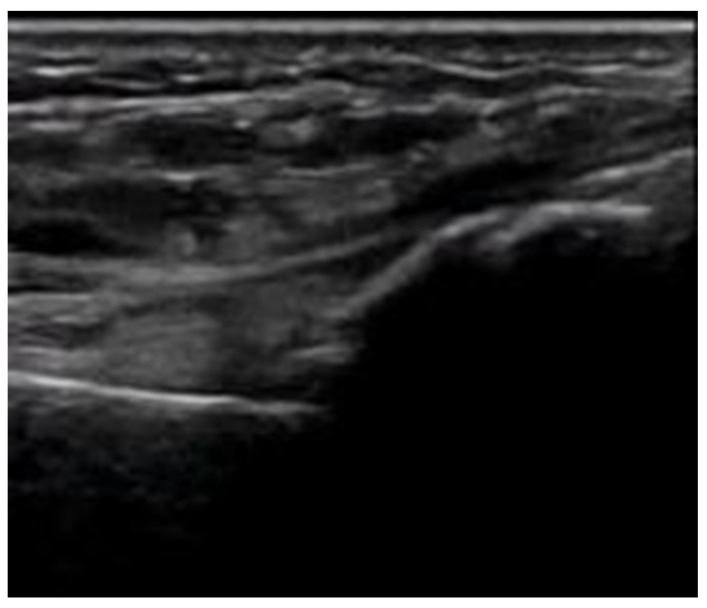
Angulated radial fracture with disruption of the corticalis.

**Figure 9:**
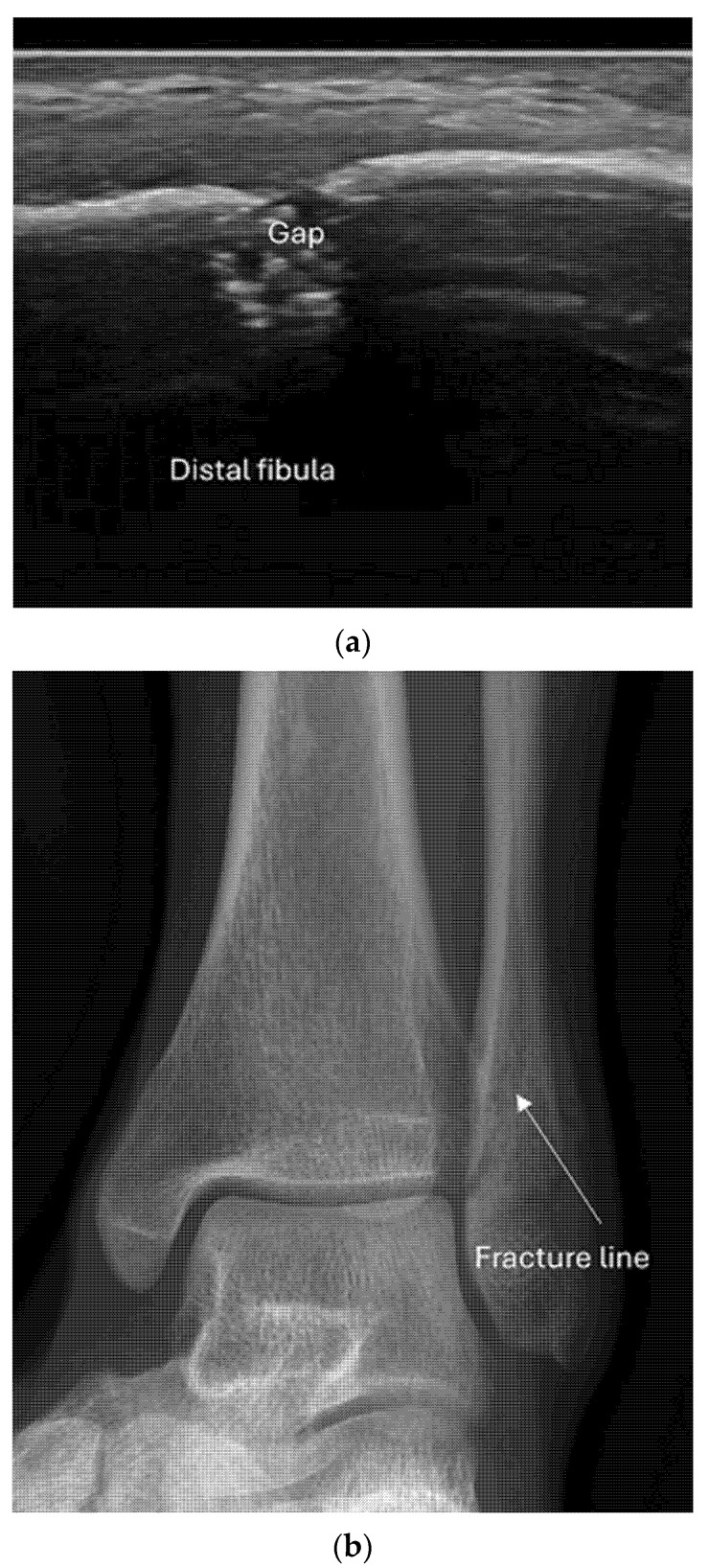
Ottawa-positive patient. (**a**) Ultrasound with lateral ankle fracture (gap). (**b**) Stable type B fracture. The fracture line is shown by arrow.

**Figure 10:**
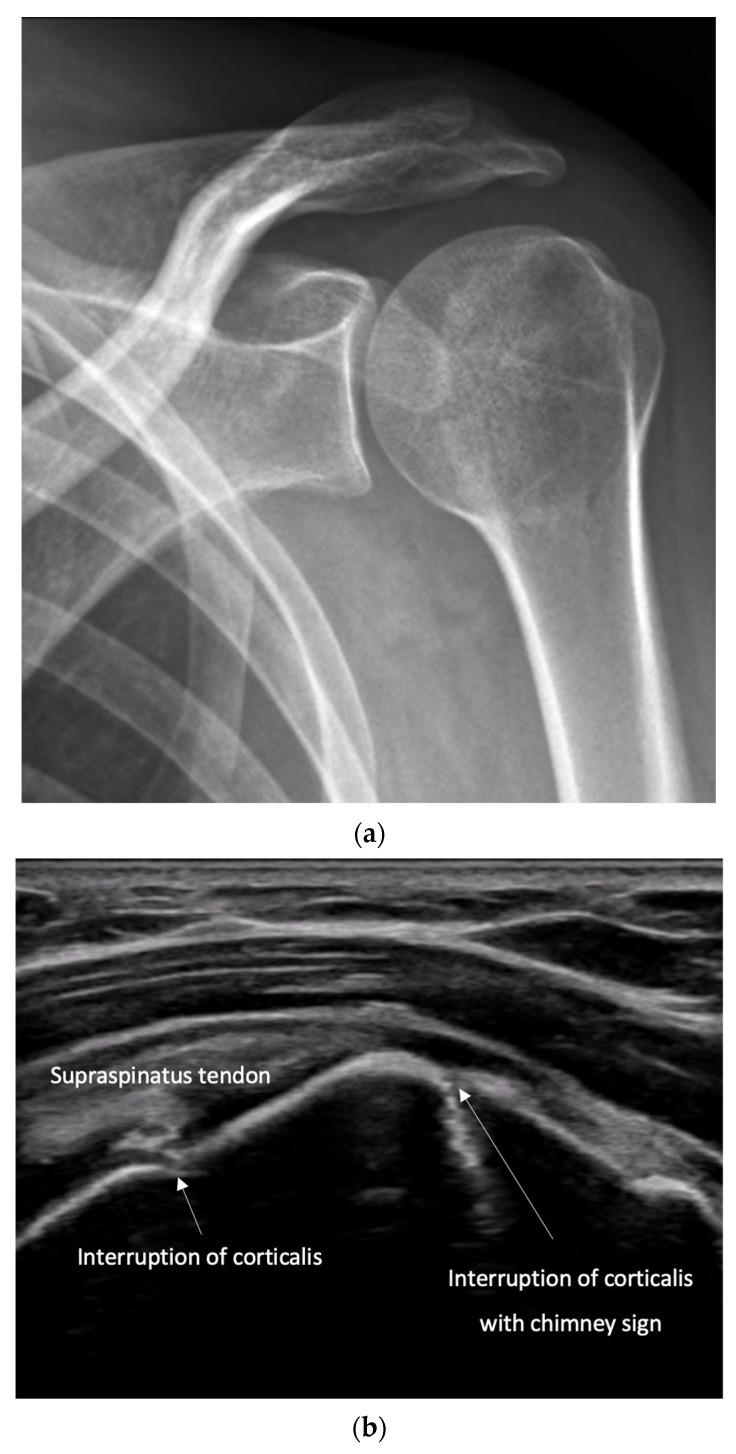
Direct trauma to shoulder. (**a**) X-ray with no clear indication of a fracture. (**b**) Non-displaced fracture of the greater tuberosity on ultrasound.

## Data Availability

Not applicable.
